# The anti‐CRISPR protein AcrIE8.1 inhibits the type I‐E CRISPR‐Cas system by directly binding to the Cascade subunit Cas11

**DOI:** 10.1002/1873-3468.70201

**Published:** 2025-10-25

**Authors:** Young Woo Kang, Hyun Ho Park

**Affiliations:** ^1^ College of Pharmacy Chung‐Ang University Seoul Korea; ^2^ Department of Global Innovative Drugs Graduate School of Chung‐Ang University Seoul Korea

**Keywords:** AcrIE8.1, anti‐CRISPR proteins, CRISPR‐Cas system, crystal structure, gene editing

## Abstract

CRISPR‐Cas systems provide adaptive immunity to bacteria by recognizing and destroying foreign genetic elements. The type I‐E CRISPR‐Cas system utilizes a multi‐subunit Cascade complex to detect target DNA and recruit the Cas3 nuclease for degradation. To overcome this defense, bacteriophages have evolved anti‐CRISPR (Acr) proteins that inhibit various steps of the CRISPR interference pathway. Here, we determined the crystal structure of AcrIE8.1, an uncharacterized Acr, revealing it binds to Cas11, a Cascade subunit, to disrupt function. AcrIE8.1 has a compact fold with a defined Cas11‐binding interface, suggesting a unique inhibitory mechanism among AcrIE proteins. These findings highlight Cas11 as a critical target for Acr‐mediated immune evasion.

**Impact statement**

Through a combination of structural and biochemical analyses, we demonstrate that AcrIE8.1 directly binds to the Cas11 subunit of the Cascade complex to inhibit the CRISPR‐Cas system. This represents a novel inhibitory strategy not previously observed among AcrIE proteins.

## Graphical abstract

In this study, we present the structure of AcrIE8.1, a previously uncharacterized anti‐CRISPR protein that inhibits the type I‐E CRISPR‐Cas system. Through a combination of structural and biochemical analyses, we demonstrate that AcrIE8.1 directly binds to the Cas11 subunit of the Cascade complex to inhibit the CRISPR‐Cas system. This represents a novel inhibitory strategy not previously observed among AcrIE proteins.

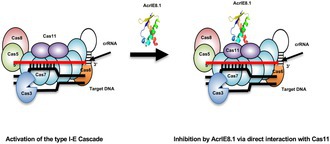

## Abbreviations

**Acr**, anti‐CRISPR

**Cas**, CRISPR‐associated

**Cascade**, CRISPR‐associated complex for antiviral defense

**CRISPR**, clustered regularly interspaced short palindromic repeats

**EMSA**, electrophoretic mobility shift assays

**IPTG**, isopropyl β‐D‐1‐thiogalactopyranoside

**MALS**, multi‐angle light scattering

**MR**, molecular replacement

**SDS/PAGE**, sodium dodecyl sulfate polyacrylamide gel electrophoresis

**SEC**, size‐exclusion chromatography

Clustered regularly interspaced short palindromic repeats (CRISPR) and CRISPR‐associated (Cas) proteins constitute a prokaryotic adaptive immune system that defends against invading genetic elements such as bacteriophages and plasmids [1, 2, 3, 4, 5]. Among the diverse CRISPR‐Cas types, the type I‐E system is one of the most extensively studied, particularly in *Escherichia coli* [6]. This system employs a multi‐subunit surveillance complex known as Cascade (CRISPR‐associated complex for antiviral defense), which recognizes foreign DNA sequences via crRNA‐guided targeting and recruits the Cas3 nuclease‐helicase for DNA degradation [2, 6, 7].

To counteract CRISPR‐mediated bacterial immunity, bacteriophages have evolved anti‐CRISPR (Acr) proteins that inhibit diverse CRISPR‐Cas systems through a variety of mechanisms [8, 9, 10, 11]. Through a combination of computational prediction and experimental validation, nearly 100 anti‐CRISPR (Acr) genes have been discovered in phage genomes [12, 13]. Owing to their minimal sequence similarity, these Acrs are categorized according to the CRISPR‐Cas subtypes they antagonize [12, 14, 15]. In type I‐E CRISPR‐Cas systems, several AcrIE proteins have been identified, each employing distinct mechanisms to inhibit Cascade activity. For example, AcrIE1 directly binds to Cas3 and blocks its recruitment to the Cascade complex [16]. Although the precise mechanism of AcrIE2 remains unclear, structural studies suggest that it may directly associate with the Cascade complex and inhibit Cas3 recruitment rather than interfering with DNA binding [17, 18]. Additionally, AcrIE4 has been shown to inhibit type I‐E systems by binding to Cas8 [19]. Most recently, a study revealed that AcrIE7 suppresses CRISPR interference by binding directly to the R‐loop formed during Cascade activation, thereby preventing Cas3‐mediated cleavage of the target DNA [20].

Despite the growing number of AcrIE proteins reported, the molecular mechanisms underlying many of these inhibitors remain poorly understood. Here, we report the high‐resolution crystal structure of AcrIE8.1, a previously uncharacterized member of the AcrIE family, and reveal its unique mechanism of CRISPR‐Cas inhibition. Biochemical and structural analyses demonstrate that AcrIE8.1 directly interacts with Cas11, thereby disrupting the function of the Cascade complex. This interaction might prevent proper Cascade assembly or R‐loop stabilization, ultimately blocking DNA targeting and Cas3 recruitment. Our findings provide mechanistic insights into a novel mode of Cascade inhibition and expand the repertoire of strategies employed by phages to suppress CRISPR‐Cas immunity.

## Methods

### Cloning, overexpression, and purification

The *acrIE8.1 gene* from *Klebsiella pneumoniae* (accession number: WP_038434996.1) was synthesized by Bionics (Daejeon, Republic of Korea) and subcloned into the pET21a expression vector (Novagen, WI, USA) using *NdeI* and *XhoI* restriction sites. The resulting construct was transformed into *Escherichia coli* BL21(DE3) competent cells. Transformed cells were cultured in 1 L of lysogeny broth supplemented with 100 μg/mL ampicillin at 37 °C. Upon reaching an optical density at 600 nm (OD₆₀₀) of 0.7–0.8, the culture was cooled to 20 °C, and protein expression was induced by adding 0.5 mm isopropyl β‐D‐1‐thiogalactopyranoside (IPTG). Induction proceeded for 18 h at 20 °C with shaking.

Cells were harvested by centrifugation at 2000 ***g*** for 15 min at 4 °C, resuspended in lysis buffer (20 mm Tris/HCl pH 8.0 and 500 mm NaCl), and lysed via ultrasonication. The lysate was clarified by centrifugation at 10 000 ***g*** for 30 min at 4 °C. The resulting supernatant was incubated with Ni‐NTA resin for 2 h at 4 °C and loaded onto a gravity‐flow column (Bio‐Rad, Hercules, CA, USA). After washing with 50 mL of washing buffer (20 mm Tris/HCl pH 8.0, 10 mm imidazole, and 500 mm NaCl), bound proteins were eluted using 2 mL of elution buffer containing 20 mm Tris/HCl pH 8.0, 500 mm NaCl, and 250 mm imidazole. Eluted fractions were further purified by size‐exclusion chromatography (SEC) on a Superdex 200 10/300 GL column (GE Healthcare, Waukesha, WI, USA) pre‐equilibrated with SEC buffer containing 20 mm Tris/HCl pH 8.0 and 150 mm NaCl. Peak fractions were pooled and concentrated to 11.3 mg/mL for crystallization and biochemical assays. Protein purity was confirmed by SDS/PAGE.

### Crystallization and X‐ray diffraction data collection

The crystallization of AcrIE8.1 was carried out using the hanging‐drop vapor diffusion method at 20 °C. For initial screening, 1 μL of protein solution (11.3 mg/mL SEC buffer) was mixed with 1 μL of reservoir solution containing 2.1 m DL‐malic acid pH 7.5 and equilibrated against 0.5 mL of the same reservoir solution. Crystals suitable for diffraction appeared within 3 days under these conditions, and further optimization confirmed that 2.1 M DL‐malic acid (pH 7.0) yielded the highest‐quality crystals.

A single crystal was selected and cryoprotected by soaking in the reservoir solution before flash‐cooling in liquid nitrogen. X‐ray diffraction data were collected at −178 °C on beamline BL‐5C at the Pohang Accelerator Laboratory (Pohang, Korea). Data processing, including indexing, integration, and scaling, was performed using HKL2000 [21].

### Structure determination and refinement

The structure of AcrIE8.1 was solved by molecular replacement using PHASER within the PHENIX software suite [22]. An AlphaFold2‐predicted structural model was employed as the search model. Initial model building was performed automatically using AutoBuild in PHENIX [23]. Subsequent model building and refinement were conducted iteratively using Coot [23] and phenix.refine [23]. The structure quality and stereochemistry were validated using MolProbity [24]. All structural figures were generated using the PyMOL program [25].

### Multi‐angle light scattering (MALS) analysis

The absolute molecular weight of AcrIE8.1 in solution was determined by SEC coupled with multi‐angle light scattering (SEC‐MALS). Purified protein was loaded onto a Superdex 200 Increase 10/300 GL column (24 mL, GE Healthcare) pre‐equilibrated with SEC buffer. Chromatography was performed at a flow rate of 0.4 mL/min at 20 °C. The eluent was monitored using a DAWN‐TREOS multi‐angle light scattering detector (Wyatt Technology) connected in‐line with an ÄKTA Explorer system (GE Healthcare). Bovine serum albumin was used as a standard for system calibration. Data acquisition and analysis were performed using ASTRA software (Wyatt Technology, Santa Barbara, CA, USA).

### Electrophoretic mobility shift assay (EMSA)

Electrophoretic mobility shift assays (EMSA) were performed to assess the nucleic acid‐binding activity of AcrIE8.1. Purified AcrIE8.1 was prepared at various concentrations (0–10 μM) and incubated with 40 nm double‐stranded DNA (dsDNA), 100 nm single‐stranded DNA (ssDNA), or 150 nm sgRNA in binding reactions. The mixtures were pre‐incubated at 4 °C for 1 h. Following incubation, samples were resolved on a 10% native polyacrylamide gel prepared with 0.5× TBE buffer. Electrophoresis was conducted at 100 V for 60 min. Gels were subsequently stained with SYBR Gold (Invitrogen, Waltham, MA, USA) and visualized according to the manufacturer's protocol.

### Pull‐down assays

Pull‐down assays were performed to assess complex formation between AcrIE8.1 and various Cascade component proteins, including Cas3, Cas5, Cas7, Cas8, and Cas11. Tag‐free AcrIE8.1 and his‐tagged Cas components were co‐transformed, and the resulting protein complex was captured on Ni‐NTA resin. The protein‐resin complexes were loaded into a gravity‐flow column (Bio‐Rad, Hercules, CA, USA) and washed with 100 mL of washing buffer to remove any unbound proteins. Bound proteins were eluted with 200 μL of elution buffer, and the co‐eluted tag‐free AcrIE8.1 was visualized by SDS/PAGE.

### SEC assay for detecting the protein complex

SEC was employed to investigate complex formation between AcrIE8.1 and Cas11 protein. For the pull‐down assay, tag‐free AcrIE8.1 and His‐tagged Cas11 were co‐transformed, and the resulting protein complex was captured on Ni‐NTA resin, allowing AcrIE8.1 to bind to Cas11. The eluted solution containing both AcrIE8.1 and Cas11 was then concentrated to 10 mg/mL and subsequently loaded onto a SEC column (GE Healthcare), pre‐equilibrated with SEC buffer containing 20 mm Tris/HCl (pH 8.0) and 150 mm NaCl. Eluted fractions corresponding to major peaks were collected and analyzed by SDS/PAGE, followed by staining with Coomassie Brilliant Blue. Co‐elution and co‐migration of protein bands were examined to assess the formation of stable complexes between AcrIE8.1 and the Cas11 proteins.

## Results and discussion

### Overall structure of AcrIE8.1 derived from *Klebsiella pneumoniae*

**Fig. 1:**
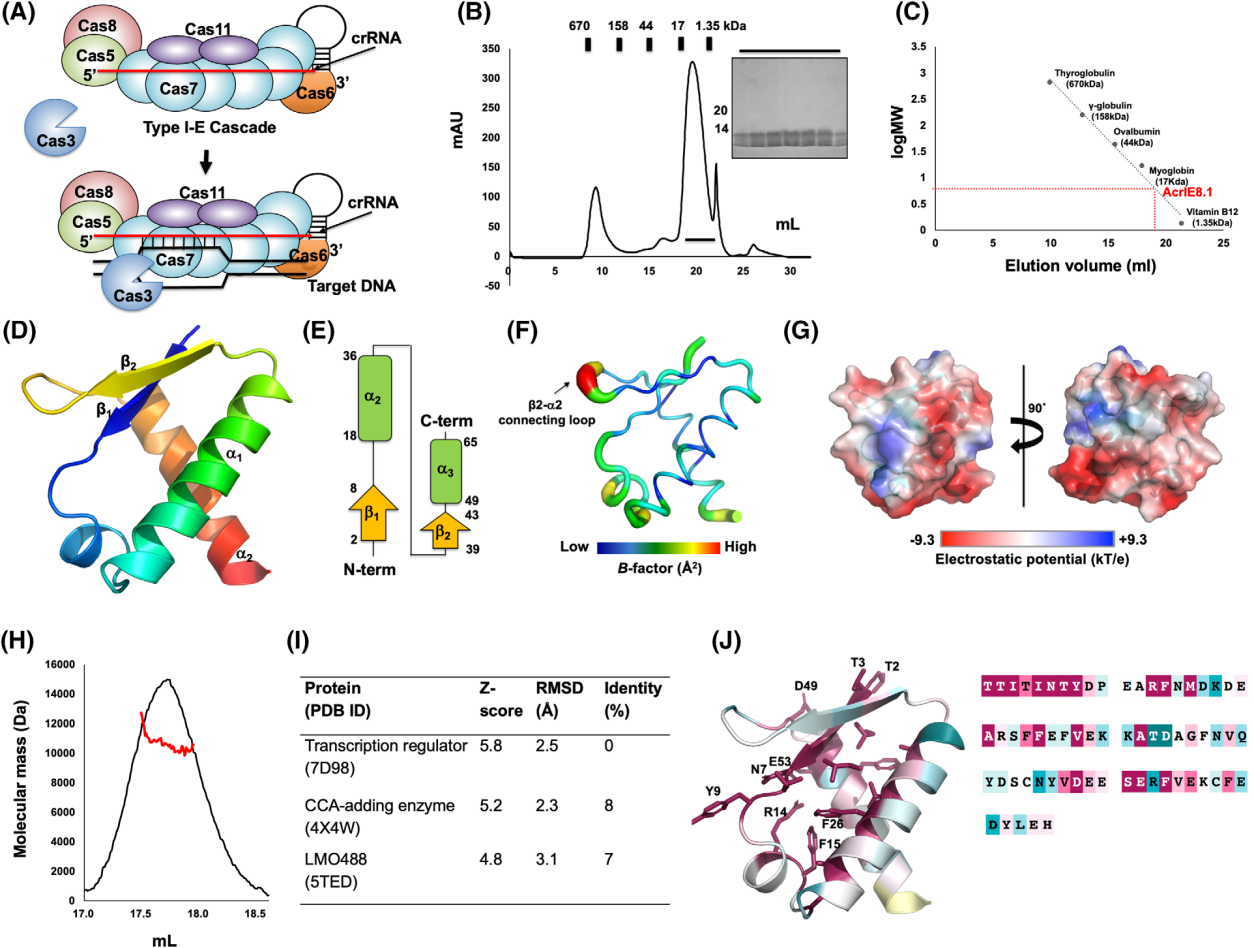
Overall structure of AcrIE8.1 derived from *Klebsiella pneumonia*. (A) An overview of the assembly and target degradation process of the Type I‐E CRISPR‐Cas system. (B) SEC profile of AcrIE8.1. SDS/PAGE gel showing the protein eluted at the peak position was provided. (C) The elution volume line fitting in SEC versus the size marker and AcrIE8.1 molecular weight logarithm. The red point on the fitting line indicates the elution volume. (D) Illustration of the structure of AcrIE8.1 presented with a crystallographic asymmetric unit. The rainbow color scheme was used for tracing the N‐ to C‐terminus. Helices and sheets are labeled with α and β, respectively. The illustration was generated by PyMol. (E) Topology diagram of the structure of AcrIE8.1. (F) *B*‐factor distribution in the structure of AcrIE8.1. The structure is presented in a putty representation and rainbow colors from red to violet in *B*‐factor value order. The highest *B*‐factor region is indicated by a black arrow. This putty representation was generated by PyMol. (G) Surface electrostatic potential of AcrIE8.1. The respective surface electrostatic distributions are represented. The scale ranges from −9.3 kT/e (red) to +9.3 kT/e (blue). This surface figure was produced by PyMol. (H) MALS profile corresponding to the main peak of the SEC. The experimental MALS data (red line) are plotted as the SEC elution volume (x‐axis) versus absolute molecular mass (y‐axis) distributions on the SEC chromatogram (black) at 280 nm. (I) Table summarizing the result of a DALI search of the PDB (RMDS = root mean square deviation). (J) Graphic representation of AcrIE8.1 colored relative to the amino‐acid sequence conservation degree generated by the ConSurf server. The completely conserved residues are labeled. This cartoon was generated by PyMol.

In the type I‐E CRISPR‐Cas system, multiple Cas proteins—including Cas5, Cas6, Cas7, Cas8, and Cas11—assemble into an RNA‐guided multi‐subunit surveillance complex known as Cascade (Fig. 1A) [26]. Upon recognition of target DNA complementary to the crRNA, Cascade recruits the trans‐acting nuclease Cas3, which mediates target DNA degradation (Fig. 1A). Type I CRISPR‐Cas systems are classified into six subtypes (I‐A to I‐F) based on the composition of their Cascade subunits [26]. Correspondingly, anti‐CRISPR proteins that inhibit type I systems are grouped into families (AcrIA to AcrIF) according to the specific subtype they target. The AcrIE family specifically antagonizes the type I‐E system and remains one of the least characterized Acr families.

To date, ten AcrIE proteins have been identified, but high‐resolution structural information is available for only six members [27]. AcrIE1 forms a homodimer and binds directly to Cas3, blocking its interaction with Cascade and thereby preventing DNA cleavage [28]. AcrIE4 has been shown to bind Cas8; however, its precise inhibitory mechanism is still unknown [19]. For AcrIE2, structural data have been reported, but the molecular basis of inhibition remains to be elucidated [17, 18]. More recently, AcrIE7 was reported to inhibit the CRISPR‐Cas system by binding to ssDNA and thereby restricting Cas3 access [20].

To understand the inhibitory mechanism of AcrIE8.1 against type I‐E CRISPR‐Cas systems via structural and biochemical analysis, the full‐length AcrIE8.1 protein (residues 1–63) was purified using a rapid two‐step chromatography method comprising affinity chromatography followed by size‐exclusion chromatography (SEC). Based on the fact that AcrIE8.1 was eluted from the SEC column at approximately 19–20 mL, we speculated that AcrIE8.1 existed in a monomeric state in the solution (Fig. 1B,C). Following purification, the target protein was successfully crystallized, and its structure was determined at 2.68 Å resolution using molecular replacement (MR), with the AlphaFold2‐predicted model serving as the search template [29]. An overview of the crystallographic and refinement statistics is provided in Table 1.

**Table 1:** Structural data and refinement statistics of AcrIE8.1. RMSD, root‐mean‐square deviation.

Data collection	
Space group	*P 32 2 1*
Unit cell parameter *a*, *b*, *c* (Å)	
*a*, *b*, *c* (Å)	46.56, 46.56, 62.84
*α, β, γ* (°)	90, 90, 120
Resolution range (Å)^1^	23.28–2.68 (2.77–2.68)
Total reflections	45230
Unique reflections	2400
Multiplicity	18.8 (19.2)
Completeness (%)^a^	99.75 (100.00)
Mean *I*/σ(*I*)^a^	45.05 (25.44)
*R*_merge_ (%)^a^, ^b^	0.06641 (0.1474)
Wilson *B*‐factor (Å^2^)	23.11
Refinement	
Resolution range (Å)	23.28–2.68 (2.779–2.68)
Reflections	2400
*R*_work_ (%)	0.1923 (0.1764)
*R*_free_ (%)	0.2776 (0.3005)
No. of molecules in the asymmetric unit	1
No. of non‐hydrogen atoms	559
Macromolecules	547
Solvent	12
Average *B*‐factor values (Å^2^)	26.46
Macromolecules	26.49
Solvent	25.04
Ramachandran plot:	
Favored/allowed/outliers (%)	100.00/0.00/0.00
Rotamer outliers (%)	0
Clashscore	5.82
RMSD bonds (Å)/angles (°)	0.004/0.65

^a^ Values for the outermost resolution shell in parentheses.

^b^ *R*_merge_ = ∑_*h*_ ∑_*i*_|*I*(*h*)_*i*_ − <*I*(*h*)>|/∑_*h*_ ∑_*i*_ *I*(*h*)_*i*_, where *I*(*h*) is the observed intensity of reflection *h*, and <*I*(*h*)> is the average intensity obtained from multiple measurements.

A single molecule of AcrIE8.1 was present in the crystallographic asymmetric unit (ASU), and the final model encompassed residues T2 to H66 (Fig. 1D). Three extra residues (LEH) at the C terminus, which were derived from the expression vector, were observed and included in the final model. The overall structure adopts a compact helix and sheet mixed fold composed of two α‐helices and two β‐sheets (Fig. 1D,E). *B*‐factor analysis indicated that the protein structure is generally rigid, with a low average *B*‐factor of 24.5 Å^2^, except for the β2‐α1 connecting loop, which exhibited elevated *B*‐factors averaging 54.3 Å^2^ (Fig. 1F).

To gain insights into the potential inhibitory mechanism of AcrIE8.1, we next analyzed its surface polarity, as surface electrostatics often play a critical role in the function of proteins with unknown mechanisms. Electrostatic surface mapping revealed that the surface of AcrIE8.1 is predominantly acidic, with two distinct basic patches observed (Fig. 1G). Proteins with such surface characteristics often do not bind directly to nucleotides, but rather interact with the Cascade complex by mimicking negatively charged molecules such as crRNA or DNA [30, 31, 32]. This suggests that AcrIE8.1 may function in a similar manner, possibly binding to the Cascade complex through nucleotide mimicry.

Since the functional stoichiometric characteristics of Acrs, whether as a monomer or dimer, are important for understanding the molecular mechanism of action of Acrs [28, 33, 34, 35], MALS was performed for analyzing the exact stoichiometry of AcrIE8.1 by determining the absolute molecular mass of the particle in solution. MALS analysis showed that the molecular mass of AcrIE8.1 in solution was 10.75 kDa (4.8% fitting error) (Fig. 1H). Since the theoretical molecular weight of monomeric AcrIE8.1 including the C‐terminal His‐tag was 9.59 kDa, MALS‐based calculation indicated that AcrIE8.1 existed as a monomer in solution.

To further investigate the potential function of AcrIE8.1, we conducted a structural homology search using the DALI server [36]. The top three structural matches included a transcription regulator (PDB ID: 7D98), a CCA‐adding enzyme (PDB ID: 4X4W), and an LMO488 protein (PDB ID: 5TED) (Fig. 1I). However, the Z‐scores of these hits ranged from 5.8–4.8, suggesting only modest structural similarity. Moreover, the relatively high root‐mean‐square deviation (RMSD) values (2.3–3.1 Å) and low sequence identities (0–8%) indicate that these proteins are unlikely to share functional relevance with AcrIE8.1.

Finally, we performed an evolutionary conservation analysis using ConSurf [37] to identify functionally important residues. This analysis revealed that several residues—including T2, T3, N7, Y9, R14, F15, F26, D49, and E53—are highly conserved across homologs (Fig. 1J). Excluding the hydrophobic residues likely involved in maintaining protein folding, T2, T3, N7, Y9, R14, D49, and E53 are located on the protein surface and are therefore likely to play important roles in the function of AcrIE8.1, possibly through interactions with other biomolecules.

### AcrIE8.1 directly binds the Cascade subunit Cas11

**Fig. 2:**
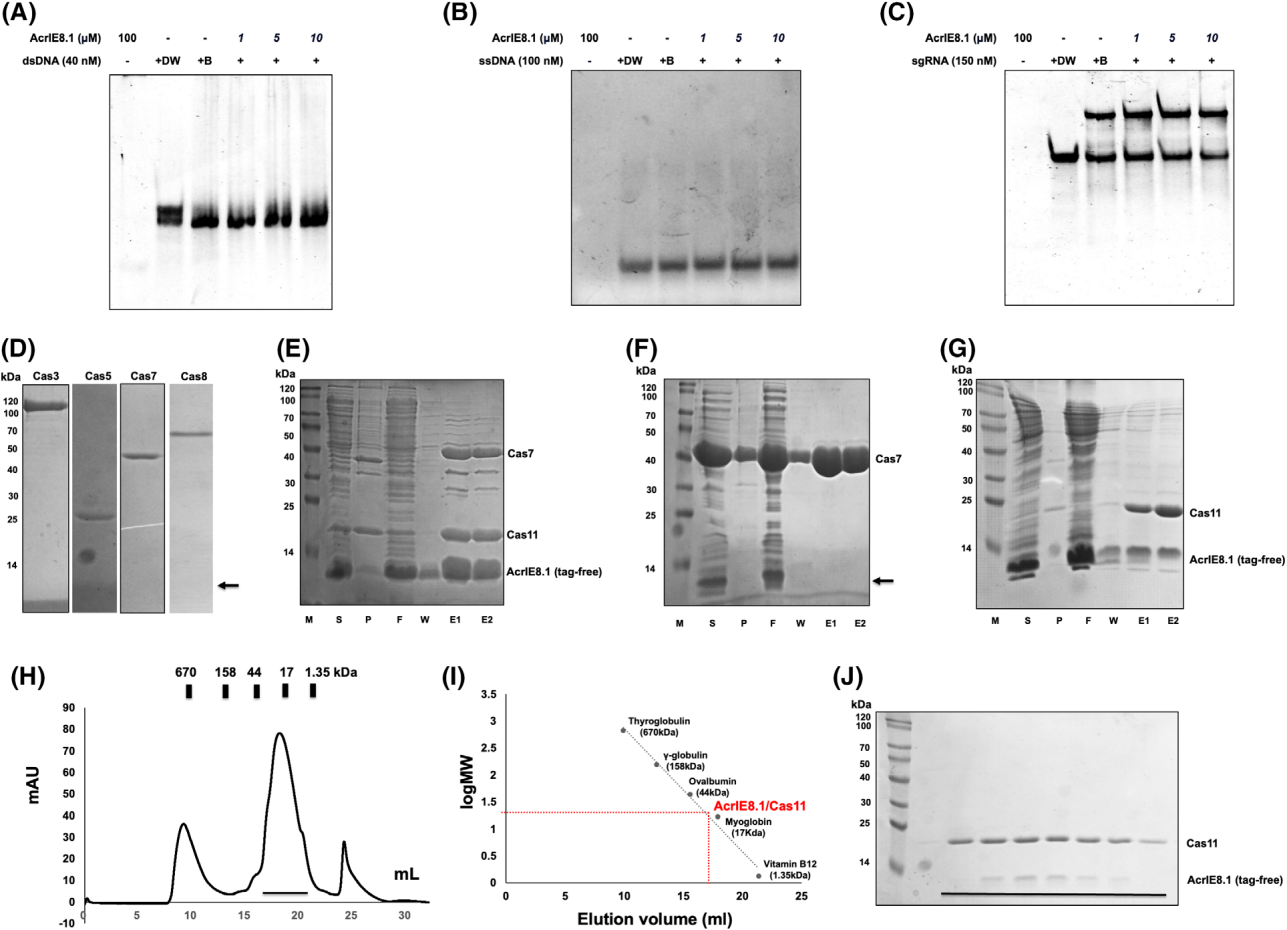
Analysis of the interaction of AcrIE8.1 with each component of the Cascade complex. (A–C) Electrophoretic mobility shift assay (EMSA) to detect the interaction between AcrIE8.1 and dsDNA (A) or ssDNA (B) or sgRNA (C). + and − indicate added and not added, respectively. DW, distilled water; B, buffer. (D) Pull‐down analysis of various Cascade components Cas protein with tag‐free AcrIE8.1. The position of tag‐free AcrIE8.1 is indicated by the black arrow. (E) Pull‐down analysis of Cas7‐Cas11 fusion protein with tag‐free AcrIE8.1. M, protein size marker; S, supernatant; P, pellet after cell lysis; F, flow‐through; W, washing; E, elution. (F) Pull‐down analysis of Cas7 protein with tag‐free AcrIE8.1. The position of tag‐free AcrIE8.1 is indicated by the black arrow. (G) Pull‐down analysis of Cas11 protein with tag‐free AcrIE8.1. (H) Size‐exclusion chromatography (SEC) profile of pull‐downed elution fractions obtained from the pull‐down assay of Cas11 protein with tag‐free AcrIE8.1. (I) The elution volume line fitting in SEC versus the size marker and AcrIE8.1/Cas11 complex molecular weight logarithm. The red point on the fitting line indicates the elution volume. (J) An SDS/PAGE gel loaded with the peak fractions of the SEC profile is provided to the right of the profile. Loaded fractions are indicated by the horizontal black bar. The corresponding fractions from SEC loaded onto the SDS/PAGE gel are indicated by the black bar. All biochemical experiments were independently repeated twice, yielding consistent results. The representative gel image shown here was selected based on clarity of presentation.

When AcrIE8.1 was first identified, it was shown to specifically inhibit the type I‐E CRISPR‐Cas system through a plaque forming assay [13, 38]. To address how a protein with the observed structure of AcrIE8.1 inhibits the CRISPR‐Cas system, we first investigated its potential to bind various nucleotides present in the system. Based on our electrostatic surface analysis, which predicted a low likelihood of direct nucleotide binding, we performed electrophoretic mobility shift assays (EMSA). Consistent with the prediction, AcrIE8.1 did not bind to dsDNA (Fig. 2A), ssRNA (Fig. 2B), or sgRNA (Fig. 2C). In the case of sgRNA, two bands were observed under buffer conditions containing salt (Fig. 2C); however, these were not due to interaction with AcrIE8.1, but rather reflected the intrinsic behavior of sgRNA under such conditions.

Since AcrIE8.1 did not bind to nucleic acids, we analyzed the direct interaction of AcrIE8.1 with each component of the type I‐E Cascade complex derived from *Pseudomonas aeruginosa* by performing pull‐down assays. For this pull‐down assay, we generated and used no‐tagged AcrIE8.1. These pull‐down assays showed that AcrIE8.1 was not pulled down with Cas3, Cas5, Cas7, and Cas8, indicating that AcrIE8.1 did not bind to those Cascade components (Fig. 2D). However, when AcrIE8.1 was co‐transformed with a Cas7‐Cas11 fusion construct, it was clearly detected in SDS/PAGE following pull‐down, indicating a physical interaction (Fig. 2E). In contrast, when only Cas7 was present, AcrIE8.1 was not co‐purified (Fig. 2F), suggesting that the interaction is specific to Cas11. Since Cas11 is known to be unstable when expressed alone but becomes stable when co‐expressed with Cas7, we initially used the Cas7‐Cas11 fusion construct for the pull‐down assay. To directly assess the interaction between AcrIE8.1 and Cas11, we attempted to express Cas11 alone. Although Cas11 was unstable and difficult to purify by itself, co‐expression with AcrIE8.1 stabilized the protein, allowing successful purification. Based on this result, we performed a pull‐down assay with co‐expressed Cas11 and AcrIE8.1, and, as expected, both proteins were co‐purified (Fig. 2G). Finally, to confirm their interaction under native conditions, we subjected the Cas11/AcrIE8.1 complex derived from the pull‐down assay to SEC. The SEC results showed a main peak eluting between the size markers ovalbumin (44 kDa) and myoglobin (17 kDa) (Fig. 2H). Based on this elution profile, we infer the potential formation of a 1:1 complex consisting of one Cas11 subunit (20.5 kDa) and one tag‐free AcrIE8.1 molecule (8.5 kDa) (Fig. 2H,I). SDS/PAGE analysis of the main elution peak revealed the presence of both proteins, demonstrating that AcrIE8.1 and Cas11 co‐migrate during SEC and thereby confirming their direct interaction (Fig. 2J).

In summary, our study concludes that AcrIE8.1, a protein with a highly novel structure, functions as a monomer that binds directly to Cas11 to inhibit the type I‐E CRISPR‐Cas system. Cas11 is known to play a critical role in stabilizing the R‐loop and maintaining the integrity of the Cascade complex [7]. Although the exact mechanism by which AcrIE8.1 inhibits Cascade through Cas11 binding remains unclear, it is plausible that AcrIE8.1 either disrupts proper R‐loop formation or interferes with the structural stabilization of the Cascade complex, thereby preventing its function. Further studies will be necessary to elucidate the detailed inhibitory mechanism of AcrIE8.1 following its interaction with Cas11.

## Conflict of interest

The authors declare no competing financial interests.

## Author contributions

HHP designed and supervised the project. YWK performed cloning, expression, and protein purification. YWK performed crystallization and collected X‐ray data. YWK and HHP analyzed the protein structure. YWK performed MALS and other biochemical assays. HHP and YWK wrote the manuscript. All authors discussed the results and approved the manuscript.

## Data Availability

Atomic coordinates and structure factors for the reported crystal structures have been deposited with the Protein Data Bank under accession number 9VX9.
